# Supplementary material for: Synthesis and Evaluation of Antifungal and Antibacterial Abilities of Carbon Nanotubes Grafted to Poly(2-hydroxyethyl methacrylate) Nanocomposites
Source: Polymers (Basel). 2023 Sep 5;15(18):3657. doi: 10.3390/polym15183657 (PMC10534391; doi:10.3390/polym15183657)
Supplement: Supplementary file 1 [file polymers-15-03657-s001.zip › polymers-2564933-supplementary.pdf]

Supplementary Material

Synthesis and Evaluation of Antifungal and Antibacterial Abilities of Carbon Nanotubes Grafted to Poly(2-hydroxyethyl methacrylate) Nanocomposites

Karina Sandoval-García [1], Abraham G. Alvarado-Mendoza [2], Eulogio Orozco-Guareño [2], María A. Olea-Rodríguez [3], Leonardo R. Cajero-Zul [4] and Sergio M. Nuño-Donlucas [4,*]

[1] Doctorado en Ciencias en Química, Centro Universitario de Ciencias Exactas e Ingenierías, Universidad de Guadalajara, Guadalajara 44430, Mexico; karina.sandoval2995@alumnos.udg.mx
[2] Departamento de Química, Centro Universitario de Ciencias Exactas e Ingenierías, Universidad de Guadalajara, Guadalajara 44430, Mexico; gabriel.alvarado@academicos.udg.mx (A.G.A.-M.); eulogio.orozco@academicos.udg.mx (E.O.-G.)
[3] Departamento de Farmacología, Centro Universitario de Ciencias Exactas e Ingenierías, Universidad de Guadalajara, Guadalajara 44430, Mexico; maria.olea@academicos.udg.mx
[4] Departamento de Ingeniería Química, Centro Universitario de Ciencias Exactas e Ingenierías, Universidad de Guadalajara, Guadalajara 44430, Mexico; leonardo.cajero@academicos.udg.mx (L.A.C.-Z.); gigio@cencar.udg.mx (S.M.N.-D.)
* Correspondence autor (S.M.N.-D.): gigio@cencar.udg.mx

Number of pages: 7
Number of figures: 12

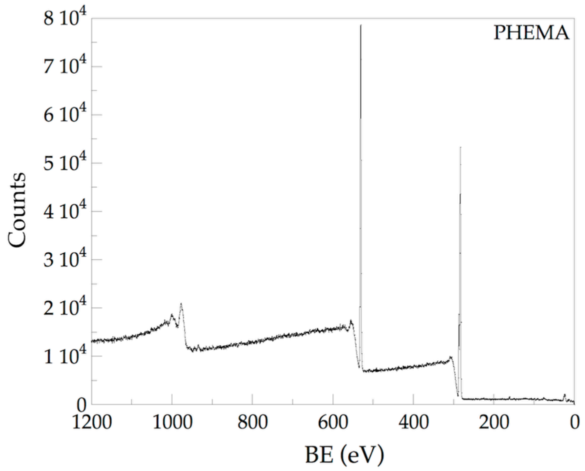

Figure S1. XPS survey of pure PHEMA.

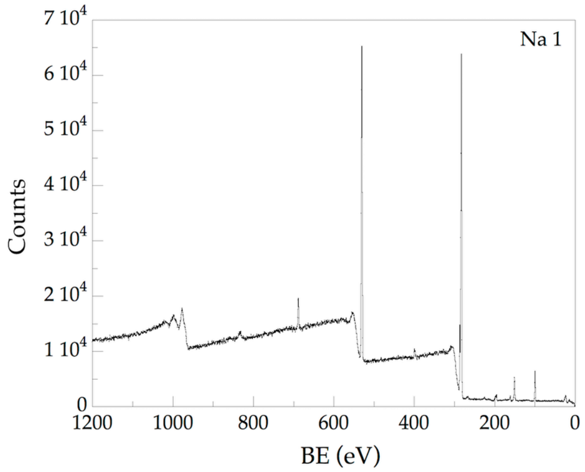

Figure S2. XPS survey of Na 1.

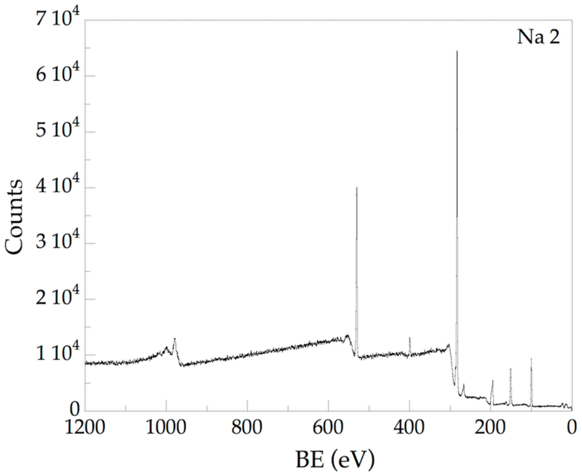

Figure S3. XPS survey of Na 2.

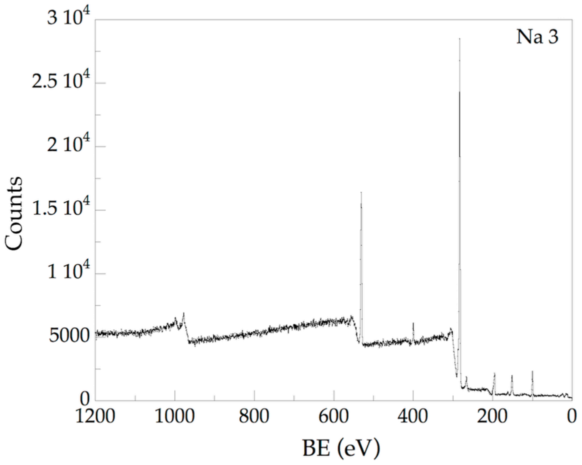

Figure S4. XPS survey of Na 3.

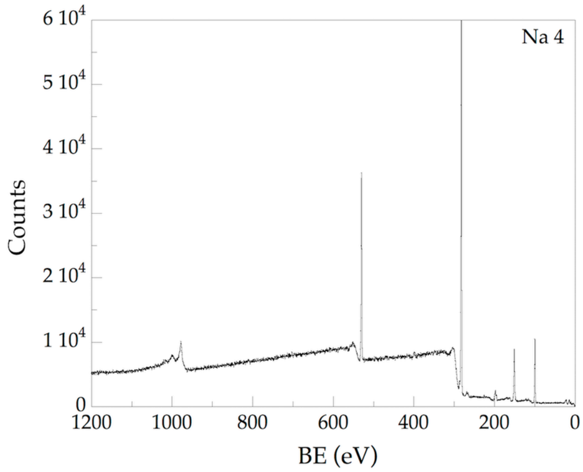

Figure S5. XPS survey of Na 4.

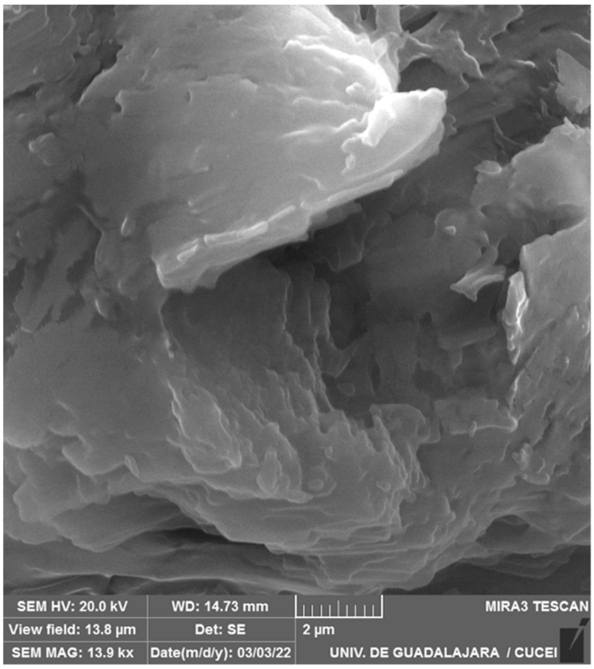

Figure S6. FE-SEM micrograph of Na 1.

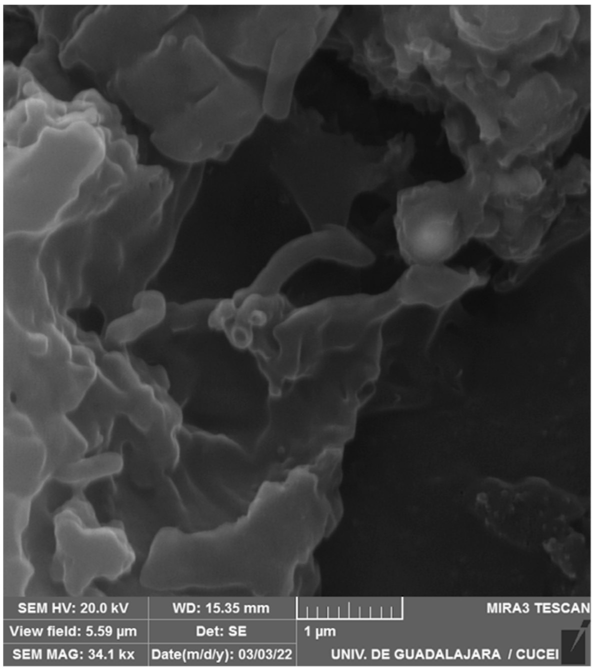

Figure S7. FE-SEM micrograph of Na 2.

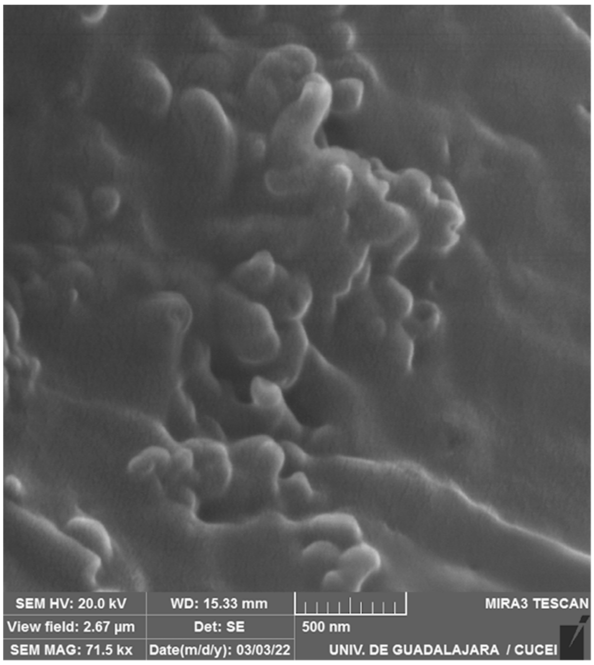

Figure S8. FE-SEM micrograph of Na 3.

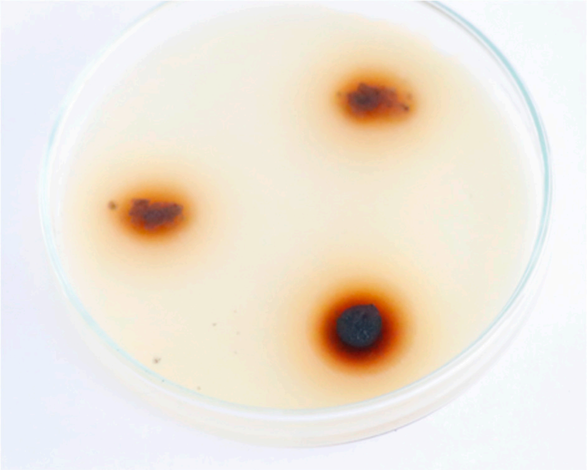

Figure S9. Photograph of a Petri dish obtained of an inhibition test of Na 1 against the growth of E. coli ATCC 25922.

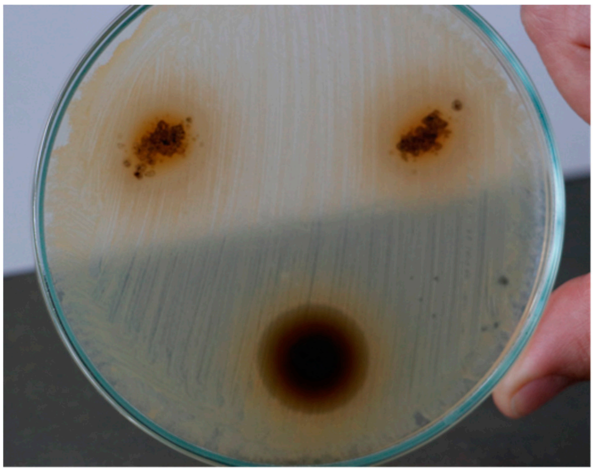

Figure S10. Photograph of a Petri dish obtained of an inhibition test of Na 2 against the growth of P. aeruginosa ATCC 9027.

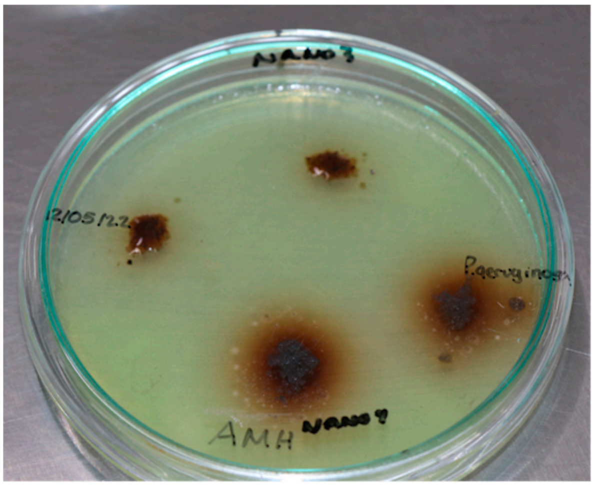

Figure S11. Photograph of a Petri dish obtained of an inhibition test of Na 3 against the growth of S. aureus ATCC 6538.

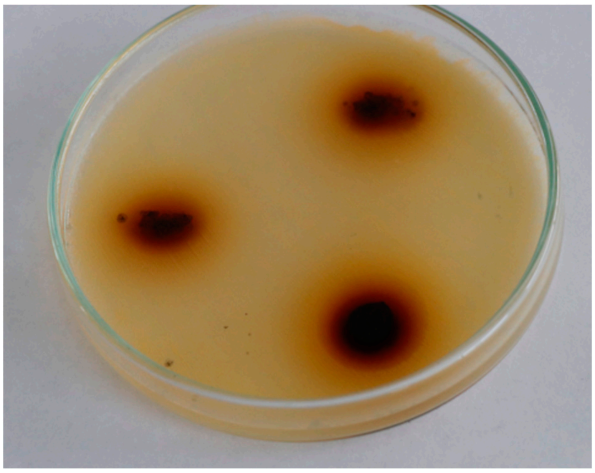

Figure S12. Photograph of a Petri dish obtained of an inhibition test of Na 4 against the growth of E. coli ATCC 25922.
